# Soft gripper for small fruits harvesting and pick and place operations

**DOI:** 10.3389/frobt.2023.1330496

**Published:** 2024-01-18

**Authors:** Eduardo Navas, Redmond R. Shamshiri, Volker Dworak, Cornelia Weltzien, Roemi Fernández

**Affiliations:** ^1^ Centre for Automation and Robotics (CAR) UPM-CSIC, Madrid, Spain; ^2^ Leibniz Institute for Agricultural Engineering and Bioeconomy (ATB), Potsdam, Germany; ^3^ Agromechatronics, Technische Universität Berlin, Potsdam, Germany

**Keywords:** 3D printing, agriculture 4.0, fruit harvesting, grasping, gripper, robotic device, robotic manipulation, soft robot

## Abstract

Agriculture 4.0 presents several challenges for the automation of various operations, including the fundamental task of harvesting. One of the crucial aspects in the automatic harvesting of high value crops is the grip and detachment of delicate fruits without spoiling them or interfering with the environment. Soft robotic systems, particularly soft grippers, offer a promising solution for this problem, as they can operate in unstructured environments, manipulate objects delicately, and interact safely with humans. In this context, this article presents a soft gripper design for harvesting as well as for pick-and-place operations of small and medium-sized fruits. The gripper is fabricated using the 3D printing technology with a flexible thermoplastic elastomer filament. This approach enables the production of an economical, compact, easily replicable, and interchangeable gripper by utilizing soft robotics principles, such as flexible structures and pneumatic actuation.

## 1 Introduction

Agriculture 4.0 is a rapidly developing field that utilizes advanced technologies such as robotics, artificial intelligence, and the Internet of Things to improve agricultural productivity, sustainability, and efficiency (Liu et al., 2020). Some of the main challenges of Agriculture 4.0, in terms of robotics, are (i) the capacity to operate in unpredictable and unstructured environments, including irregular terrains and variable weather conditions, (ii) performing a wide range of tasks such as planting, harvesting, and pest control. Each of these tasks requires specific knowledge and expertise, and developing a robotic system that can perform them all effectively is a major engineering challenge. (iii) Reliability and durability are crucial considerations when designing agricultural robots. They must be able to operate continuously for long periods with minimal maintenance. Finally, (iv) cost is another major challenge associated with the development and deployment of agricultural robots (Cheng et al., 2023). The high cost of research and development, production, and deployment of these systems can limit their adoption, especially by small farmers.

In this context, soft robotics, and particularly soft grippers, have been proposed as a promising solution to the problem of automated agriculture tasks that require manipulation capabilities (Navas et al., 2021). The main advantages that this technology can bring to agriculture are: (i) Flexibility: Soft robots are made of flexible materials that allow them to move and adapt to unstructured agricultural environments more easily than traditional rigid robots. (ii) Human interaction: Soft robots are safer to operate near humans and delicate objects since they are less likely to cause damage or injuries. (iii) Versatility: Soft robots can be designed to perform a wide range of tasks, from harvesting to maintenance or pick and place operations. (iv) Durability: Soft robots can withstand impacts and deformations, making them ideal for use in difficult or unpredictable environments. This is common in agricultural applications, where machinery is exposed to significant wear and tear. (v) Cost-effectiveness: Soft robots can be made with low-cost materials and fabrication techniques, which makes them more affordable and accessible for a wider range of applications, as well as easy to repair or replace.

Therefore, these soft grippers can be a game changer for all those fruits that, either due to their difficulty, economic feasibility, or other factors, have not yet been automated. This is the case for blueberries, for which the software part has been widely investigated (Li et al., 2010; Kuzy et al., 2018), but the hardware part has not been researched as much, and attempts to automate their harvesting have had little success (Hall et al., 1983). Table 1 shows a list of all those small and medium-sized fruits for which automation still poses a challenge.

**TABLE 1:** Medium and small-sized fruits classification.

Type of fruit	Name	Actual harvesting method	Automatic harvesting method
Drupes	Blackberry	2	1 Crandall (1995)
	Cherry	2	1 Halderson (1966); Norton et al. (1962); Peterson and Wolford (2001); Zhou et al. (2012), Chen et al. (2012a), Zhou et al. (2013); He et al. (2013), 2 Tanigaki et al. (2008), * Amatya et al. (2017)
	Cafe	2	1 Santinato et al. (2016); Aristizábal et al. (2000)
	Raspberry	2	1 Patzlaff (1971), Patzlaff (1972)
Berries	Blueberry	2	1 Richard (1982), 2 Navas et al. (2023)
	Grape	1,3	1 Shaulis et al. (1966); Shepardson et al. (1969); Studer and Olmo (1969); Pezzi and Caprara (2009), 2 Monta et al. (1995), * Luo et al. (2016)
	Kiwi	2	2 Chen et al. (2012b); Mu et al. (2017); Williams et al. (2019)
	Passion fruit	2	* Tu et al. (2018)
	Wolfberry	2	1 Qiang et al. (2009), 2 Bing and Jing, (2011), * Lvcheng et al. (2013)
Hesperidium and Pepo	Lime	3	2 Nemlekar et al. (2021)
Aggregate fruit	Strawberry	2	2 Rajendra et al. (2009); Hayashi et al. (2010); Dimeas et al. (2015); De Preter et al. (2018); Klaoudatos et al. (2019); Huang et al. (2020)
Multiple fruit	Fig	2	-

1 Mechanical motion towards the fruit indirectly via force exerted on the plant itself.

2Application of a mechanical force directly onto the fruit or its peduncle.

3Direct mechanical motion or an alternative cutting approach implemented directly on the stem.

*Artificial Intelligence researches for fruit detection.

For this reason, this article aims to present a soft gripper design approach based on the integration of a pneumatically actuated soft diaphragm actuator and a 3D-printed flexible structure into a single compact module. Data collected by finger-tracking gloves have been used to design the gripper, which has helped to adapt the design to human movement patterns. This results in a soft gripper suitable for agricultural tasks and pick and place operations. The main novelties and contributions are:

• The study of the movement patterns involved in blueberry harvesting from data collected with finger-tracking gloves to better adapt the soft gripper.
• The design of a compact hybrid soft gripper that combines flexible structure technologies with different levels of stiffness, indirect motion through pneumatic actuation, and a rotating rigid structure that allows for various types of grip or picking patterns. The pneumatic structure is isolated from potential failure risks through puncture prevention measures.
• The development of a design that, through the use of a uniaxial motion diaphragm actuator, simplifies the complexity of controlling soft grippers.
• The development of an easily replicable, cost-effective, and replaceable actuator suitable for agricultural tasks and pick and place operations.
• The design of a gripper capable of harvesting fruits or objects found in clusters without damaging surrounding ones.

The remainder of the article is organized as follows. Section 2 details the steps followed for the design and manufacturing of the soft gripper for berries harvesting while section 3 presents the control system. Section 4 describes a testbed for measuring the characteristics of soft actuators, followed by a discussion of the main results obtained from the experimental evaluation of the proposed soft gripper. Finally, the main conclusions are summarized in Section 6.

## 2 Design and manufacturing approach

To identify the essential requirements that a soft gripper should meet to be fully operational, the agricultural processes that involve manipulation and, more particularly, the harvesting tasks (Duckett et al., 2018; Navas et al., 2020; Navas et al., 2021), as well as the pick and place operations (Blanes et al., 2011) have been reviewed. One of the recognized prerequisites for enhancing the profitability of harvesting machinery is the ability to customize the design for various crop varieties. With this objective in mind, the aim was to attain a completely parameterizable and scalable design concept. The soft actuator can be produced in diverse dimensions to yield a wide range of diameters and lengths. This characteristic enables a gripper conceived within this framework to be reconfigured for accommodating the harvesting of distinct kinds of fruits. Another demand in this domain is simplicity, leading to systems that are interchangeable and effortless to repair. This is why the design approach is founded upon a compact soft actuator featuring a flexible gripping structure, which can be swiftly manufactured using 3D printing technology. The additional requirements are more related to maintaining the quality standards of the fruit than to the harvest process itself. These requirements include preventing fruit damage, utilizing non-hazardous materials, and employing designs that prevent the spread of diseases and pests. Unfortunately, these crucial aspects were overlooked in the design of previous grippers, which used materials that could harm the fruits and featured complex designs that hindered cleaning. To address this, a combination of soft robotics technology, hygienic designs, and adjustable flexibility is used to ensure fruit and crop protection. Furthermore, the modular soft gripper is specifically designed to serve as the end effector of a robotic manipulator (Sepúlveda et al., 2019; Navas et al., 2021). It is capable of executing nearly all the necessary harvesting movements, commonly referred to as picking patterns in the literature (Yaguchi et al., 2016; Li et al., 2019; Huang et al., 2020; Mu et al., 2020). These picking patterns encompass a range of simple actions, such as twisting, pulling, lifting, and bending, which can be combined as required.

The following section delineates the field study that was conducted to develop a design that adequately meets the needs of the harvest process. Then, the type of soft material that has been selected for the implementation of the gripper is described highlighting its main advantages. Subsequently, the design specifications for both the rigid and soft components of the gripper is presented, with the latter having been modelled using finite element analysis. Finally, a detailed description of the manufacturing and assembly process of the gripper is provided.

### 2.1 Picking pattern study

During the design process, finger-tracking gloves have been used to gain a better understanding of the movement patterns involved in the harvesting process and consequently use this knowledge to better adapt the soft gripper design to these human movements. The utilization of finger-tracking gloves constitutes a novel approach in investigating fruit harvesting practices.

**FIGURE 1:**
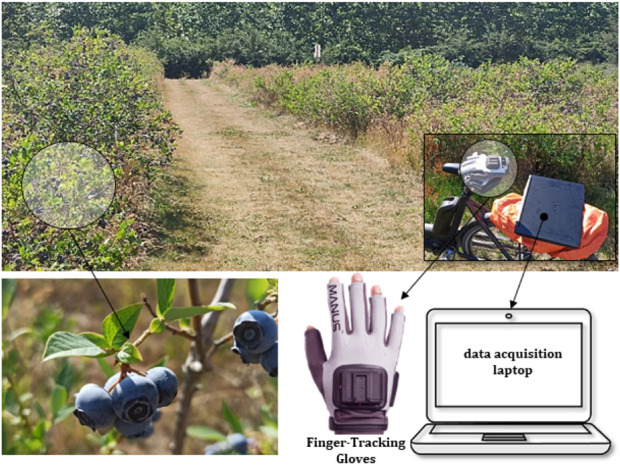
View of the Blueberry experimental field and data acquisition setup.

Conventionally, visual methodology has been the most widely employed approach in scientific literature for identifying harvesting movements (Dimeas et al., 2015; Zheng et al., 2022). However, this method is often imprecise in capturing the intricate movement patterns exhibited by human agents in fruit collection tasks. By employing finger-tracking gloves, it becomes possible to meticulously monitor the movement patterns involved in the fruit harvesting process, through numerical quantification. Consequently, this enables the detailed analysis of different harvesting patterns within a manipulation study. The experimental trials for fruit harvesting were conducted under naturalistic conditions at the Leibniz Institute of Agricultural Engineering and Bioeconomy e.V. (ATB). The chosen specimens for the experimental tests were blueberries (Vaccinium corymbosum), which were harvested from the fields of ATB Marquardt located in Potsdam, Germany. The finger-tracking gloves utilized for the data acquisition, illustrated in Figure 1, were the Manus Prime 2 (Meta, 2023), which are capable of tracking the angles between different joints, as well as the stretching angle between fingers.

**FIGURE 2:**
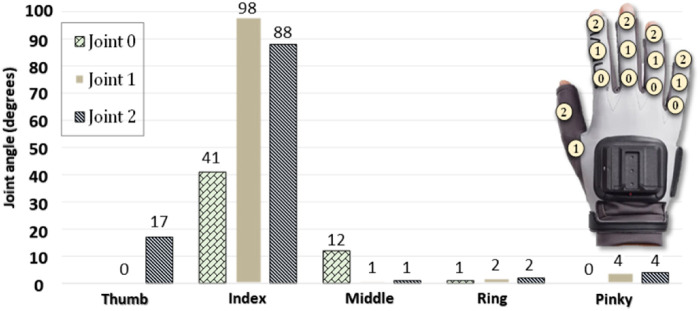
Maximum angle reached by the finger joints during blueberry harvesting (Navas et al., 2023).

To evaluate the characteristics of blueberries, a sample of 20 berries was chosen at random. The average diameter of the berries was 13 mm, with a range of 10–15 mm. The average weight was 1 g, with the weight of individual berries ranging from 0.6 to 1.4 g. Throughout the harvesting of these 20 samples, the monitoring of finger joint angles was carried out, as illustrated in Figure 2. The thumb and index finger were predominantly utilized for manipulations employing a pulling picking pattern. Quick analysis was performed using the maximum values of the joints to determine the involved fingers. The spread angles for the thumb, index, middle, ring, and pinky were 39°, 0°, 0°, 0°, and 0°, correspondingly. It is worth emphasizing that these angles remained constant, with the thumb and index finger maintaining a completely stationary position, indicating heightened rigidity in the grasping motion. Based on these observations, it can be deduced that a two-point grip is well-suited for the harvesting of blueberries.

### 2.2 Soft materials

3D printing has emerged as a promising method for the fabrication of soft actuators. This technology provides a more efficient and accurate approach, reducing the incidence of manufacturing defects and enabling a higher degree of experimental reproducibility. Moreover, software-based design modifications allow for greater control over the actuator’s performance and behavior.

In the soft robotic field, Polymer-based materials such as Ecoflex (Mosadegh et al., 2014; Hsiao et al., 2020), Dragon Skin (Connolly et al., 2017; Shih et al., 2017; De Barrie et al., 2020; Wang et al., 2020), Elastosil M4601 (Mosadegh et al., 2014; Galloway et al., 2016; Seibel et al., 2020; Teeple et al., 2020), and polydimethylsiloxane (PDMS), commercially known as Sylgard 184 (Wang et al., 2016; Rodrigue et al., 2017; Shih et al., 2017; Modabberifar and Spenko, 2018; Galley et al., 2019; Hsiao et al., 2020), are commonly used due to their unique mechanical properties and ease of processing. However, the manufacturing process for soft robots using these materials is often a time-consuming and challenging endeavor, involving several stages such as mold design and intricate fitting to prevent material leakage. Moreover, the manufacturing process may suffer from interstitial bubbles and delamination, which may lead to actuator performance degradation or even failure.

On the other hand, thermoplastic elastomers (TPEs) (Liu et al., 2020; Dilibal et al., 2021) have gained significant interest in soft robotics due to their unique properties, which enable the fabrication of complex geometries that are otherwise unattainable through traditional manufacturing processes like molding. TPEs, which are a type of polymeric material, possess elastomeric behavior and thermoplastic processability, making them ideal for use in additive manufacturing techniques such as 3D printing. The ability to produce intricate shapes using TPEs has made them a promising material for the development of soft robots, which rely on compliant structures to achieve versatile and adaptive motion.

Therefore, the material selected for the implementation of the soft gripper is a 1.75 mm TPE filament manufactured by Multicomp Pro. TPEs possess unique properties, allowing the fabrication of complex geometries through 3D printing. Their elastomeric behavior and thermoplastic processability make them ideal for creating compliant soft actuators, enabling versatile and adaptive motion. TPEs also offer flexibility, durability, and biocompatibility, further enhancing their usability in the agricultural field.

### 2.3 Soft design

Once data on fruit characteristics and picking patterns have been collected, design requirements are determined. Pattern analysis, along with other criteria such as simplicity, avoidance of fruit bruising, and the ability to harvest clustered fruit, define the design constraints. Ultimately, the soft gripper design is conceived not only as an end effector, but also as a tool for a robotic arm, capable of performing almost all picking patterns required for harvesting (Navas et al., 2021).

**FIGURE 3:**
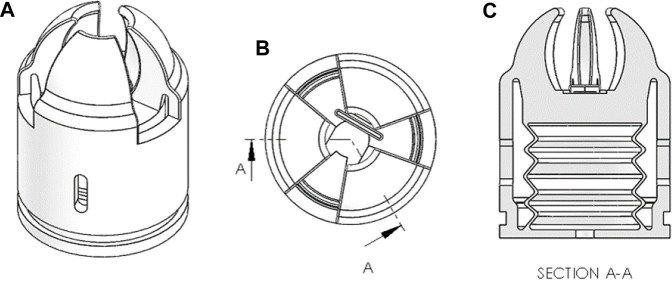
Soft Actuator. **(A)** Isometric View. **(B)** Top view. **(C)** Section view.

Regarding the geometric design, shown in Figure 3, the grippers proposed in this article consist of a single-channel diaphragm-type actuator with a flexible structure. One advantage of this design is its simplicity of manufacture, as it can be 3D printed in one piece. Another advantage is the ease of control. Soft diaphragm actuators are designed to move primarily on one axis, with negligible motion on other axes, simplifying the control of the Degrees of Freedom (DoFs) of the gripper.

The suggested flexible diaphragm also utilizes the bellows concept, which distinguishes it from other shapes in terms of how it expands. Unlike geometries based on cylinders, cubes, or spheres, where inflation typically leads to both forward elongation and wasted forces on the side walls, the bellows-based design addresses this issue. Cylindrical and cubic shapes experience reduced forward advancement due to radial expansion. Although the spherical shape performs better in terms of inflation behavior, it requires complex molding for manufacturing. However, the proposed combination of cylindrical and spherical shapes in the bellows-cylinder design partially solves this problem. It leverages the forces generated by radial inflation to achieve forward elongation, extending the gripper’s body in that direction.

Regarding the actuator structure, the finger-tracking glove data indicates that a two-point grip is well-suited for the harvesting of blueberries. However, it is important to emphasize that the design objective goes beyond optimizing the gripper for a specific fruit. The intention is to create a gripping mechanism with a more generalized design capable of not only adapting to other small and medium-sized fruit, but also performing a wide range of picking motions, including pulling, twisting, and bending the fruit peduncle, while maintaining a strong grip. Consequently, the proposed gripper comprises three contact elements that contribute to secure gripping and manipulation. While the two-points grip may be optimal for blueberries, the inclusion of a third grip point provides additional versatility for handling fruits of different shapes and sizes. Moreover, the three contact elements are reinforced by a thicker TPE wall compared to the diaphragm, ensuring a stable support point for the opening and closing actions of the gripper.

Finally, the choice of a more generic gripper aligns with the practical challenges faced in agricultural settings where a robot may encounter a high variability during harvesting or pick-and-place operations. A gripper designed for versatility can adapt to different scenarios without the need for frequent reconfigurations or specialized attachments.

**FIGURE 4:**
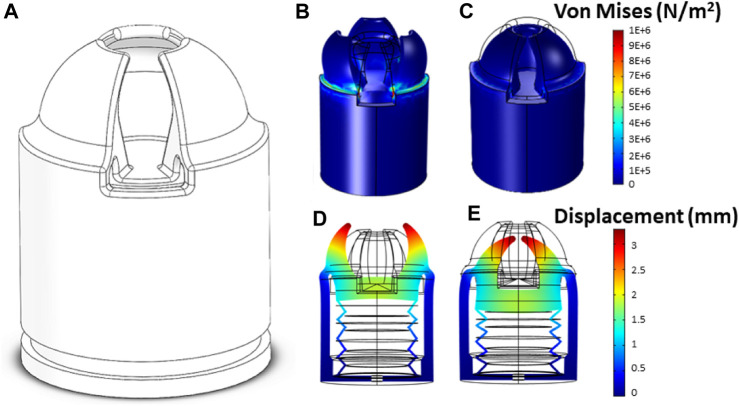
Model showing the displacement reached on the soft gripper in **(A)** normal position, **(B)** deformation in open position, **(C)** deformation in closed position, **(D)** deformation in open position and **(E)** deformation in close position.

With the aim of devising suitable soft grippers for small-sized fruits, the COMSOL Multiphysics^®^ platform is employed to model its inflation behavior through Finite Element Method (FEM), as shown in Figure 4. To accomplish this, TPE is simulated as a hyperelastic material. In existing literature, numerous mathematical frameworks exist to describe the behaviour of such 3D printed thermoplastic elastomers. Among these, the five-order Ogden model, in contrast to Yeoh’s, Van der Waals’s, or Arruda-Boyce’s models, provides a more precise depiction of its response (Adams et al., 2019).

Furthermore, as a result of the inflation pressure, the soft diaphragm will experience equibiaxial tension. This form of tension in hyperelastic materials, applicable to thermoplastic elastomers, was theorized by Ogden (Ogden, 1972). This approach pertains to elastic solids characterized by a strain–energy function and isotropic behavior in relation to the stress-free ground state. It also assumes the solid’s incompressibility. Therefore, its formulation can be expressed as follows:

$$\sigma_{i}=\mu_{r}a_{i}^{\alpha_{r}}-p,$$ (1)

where *σ*_*i*_, $i\in \left\{1,2,3\right\}$ , represents the principal Cauchy stresses (*σ*_1_, *σ*_2_, *σ*_3_), the parameters *μ*_*r*_ and *α*_*r*_ are experimentally obtained constants, *a*_*i*_ represents the stretches (*a*_1_, *a*_2_, *a*_3_) and *p* is an arbitrary hydrostatic pressure introduced because of the incompressibility constraint. Due to the equibiaxial tension, two out of three principal stresses are equal, and the third one is zero:

$$\sigma_{2}=\sigma_{3}=\sigma ,\sigma_{1}=0$$ (2)

Moreover, the stretches can be written as follows:

$$a_{2}=a_{3}=a,$$ (3)

and due to the incompressibility assumption, it can be considered that *a*_1_ = *a* ^−2^. The substitution of the aforementioned into (Eq. 1) is as follows:

$$\sigma_{i}=\mu_{r}a^{\alpha_{r}}-p,0=\mu_{r}a^{\alpha_{r}-2}-p.$$ (4)

The elimination of *p* yields:

$$\sigma_{i}=\mu_{r}\left(a^{\alpha_{r}}-a^{\alpha_{r}-2}\right).$$ (5)

Finally, Eq. 5 is incorporated into the FEM software, alongside the values of *μ*, *α*, and bulk modulus, acquired from (Kim et al., 2011). The results obtained from the FEM software are illustrated in Figure 4 for the soft gripper. The working principle of the soft gripper is as follows. When positive pneumatic pressure is applied to the inlet of the actuator, an expansion occurs in the bellows, as seen in Figures 4B, D, resulting in an opening movement. On the other hand, when negative pneumatic pressure is applied to the actuator, the bellows collapses and a closing movement is generated, as shown in Figures 4C, E. Both movements are possible due to the flexible structure of the actuator. When establishing the working pressure range for analysis, a prioritization is made for a range that ensures an adequate working volume for small fruits while maintaining low energy consumption and sufficient gripping force for various manipulation movements. Therefore, through an iterative process, a study pressure range of 50–−50 kPa is reached.

As for the structure of the soft gripper, it has been designed in such a way that different levels of rigidity have been achieved by varying the thickness of the walls, since some must be rigid for a stable grip and other requires flexibility. Regarding the latter, the walls of the bellows are printed at 0.8 mm, with a rounding radius of 1 mm and an angle between walls of 90°.

### 2.4 Design of the rigid structure

**FIGURE 5:**
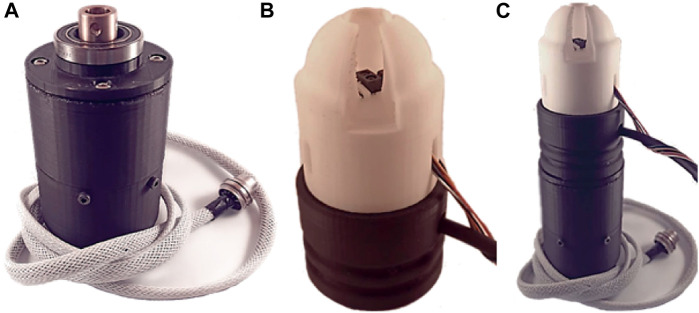
View of the **(A)** soft gripper rotating base, **(B)** soft Actuator, and **(C)** complete assembly of the soft gripper.

Following a comprehensive analysis of the collection patterns of various fruits, it was observed that a rotational motion is frequently employed to facilitate their detachment from the plant. In order to incorporate this characteristic into the designed soft gripper, a degree of freedom pertaining to rotation around the gripper’s axis was introduced. To this end, a PLA-based rotating base, as shown in Figure 5A, was integrated and actuated by a Nema 17 stepper motor.

### 2.5 Manufacturing and assembly

The fabrication of the soft gripper was carried out using Fused Filament Fabrication (FFF), in particular a Creality Ender 3 3D printer adapted to print flexible material. The printing parameters required for the proper printing of the flexible TPE filament are detailed in Appendix 1. This fabrication approach represents a promising methodology for the production of soft robotics components, as it enables the production of complex geometries and customization in a cost-effective and time-efficient manner.

The rigid components of the soft gripper were fabricated using Polylactic acid (PLA). The attachment of the soft actuator to the rigid base was accomplished using a press-fit mechanism, show in Figure 5B, avoiding the use of screws or other types of rigid fasteners. The motor was secured to the gripper claw with screws, and the coupling of the motor shaft with the movable part of the gripper was achieved through a motor coupling supported by a bearing. The lower portion of the claw is designed to be detachable, allowing it to be used as a primary soft gripper or as a soft tool for a robotic arm. Figure 5C provides a detailed illustration of the fully assembled soft gripper.

## 3 Control system

In general, soft robotics are characterized by their deformability and compliance, resulting in a large number of intrinsic DoFs. While this can increase the complexity of the control system, it also allows for a wide range of movements, including bending, twisting, stretching, compression, and buckling wrinkles (Rus and Tolley, 2015). High levels of sensorization are typically employed to tackle the soft control barrier. Alternatively, some researchers (Duriez, 2013) use real-time Finite Element Method (FEM) simulations to control soft elastomer robots. Nonetheless, for certain soft materials, establishing a reliable mathematical model can be challenging.

On the other hand, the gripper presented in this article could be seen as a specific instance of hybrid grippers (McKenzie et al., 2017; Park et al., 2020), not because it physically combines rigid components embedded in soft actuators, but because the rigid structure imposes constraints on the DoFs of the soft actuator, making it easier to control the gripper.

**FIGURE 6:**
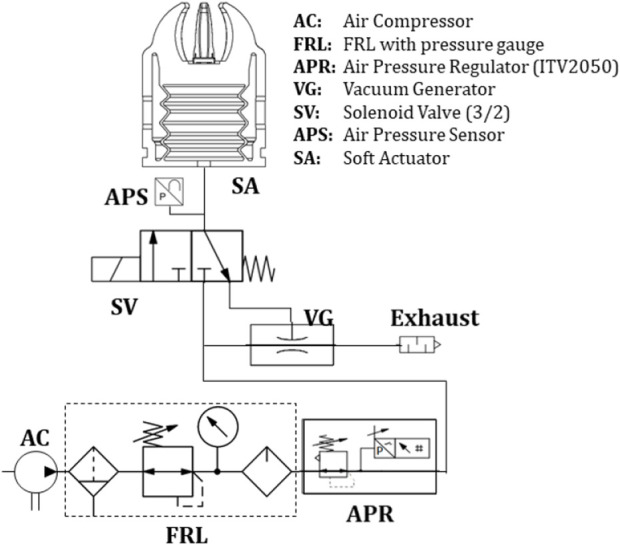
Schematic of the pneumatic control system for the soft gripper.

To operate the soft gripper effectively, it is essential to have sensor and control components that guarantee accurate air pressure measurement and a steady airflow. Figure 6 and Table 2 show the pneumatic elements schematically and their main characteristics, respectively.

**TABLE 2:** Main features of the pneumatic elements of the system.

Pneumatic element	Main characteristic	Value
Air Compressor	Compressed air deposit	6 L
	Power comsumption	1.1 kW
FRL unit	Standard nominal flow rate	1600 L/min
Pneumatic solenoid valve	Type	3/2-Way Normally Close
Air pressure regulator	Pressure range	0.005–0.9 MPa
	Power comsumption	4 W
	Max. Flow rate	1500 L/min
	Repeatability	±0.5%
	Response time	0.1 s
Air pressure sensor	Pressure range	0–100 kPa
	Measurement precision	±0.4%
Vacuum generator	Max. Vacuum pressure	80 kPa

The proposed soft gripper is then controlled by MATLAB/Simulink, enabling manual activation and adjustment of pneumatic electrovalves pressure. Moreover, the grippers can operate automatically using a proportional-integral-derivative (PID) control mechanism, in which the pressure sensor positioned at the soft actuator’s inlet provides the feedback. This allows contact detection between the soft actuator and the objects without the need for embedded sensors within it. Whilst the soft gripper has been devised for integration with a bimanual robot that employs computer vision for object detection (Sepúlveda et al., 2020), it is also integrated with an infrared sensor GP2Y0E03 (measurement range: 4–50 cm, output voltage: 2.7–5 V) that provides position feedback to the vision system, thereby assisting the execution of successful grasping. Algorithm 1 summarizes the different steps described above.

**FIGURE 7:**
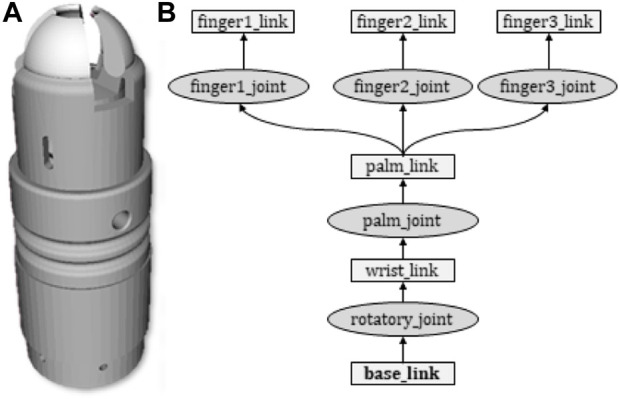
URDF model of the soft gripper implemented in ROS. **(A)** Soft gripper displayed in RViz; and **(B)** URDF specification. The links and joints are visualized by boxes and ellipses, respectively.

A Unified Robotics Description Format (URDF) model of the soft gripper has also been implemented in Robot Operating System in order to facilitate its integration with the dual-arm robotic platform and to enable the communication of the robotic planning module with the low level controller of the soft gripper. Figure 7 shows the soft gripper in the 3D visualization program RVIZ.

Algorithm 1 Algorithm to address soft gripper grasping.

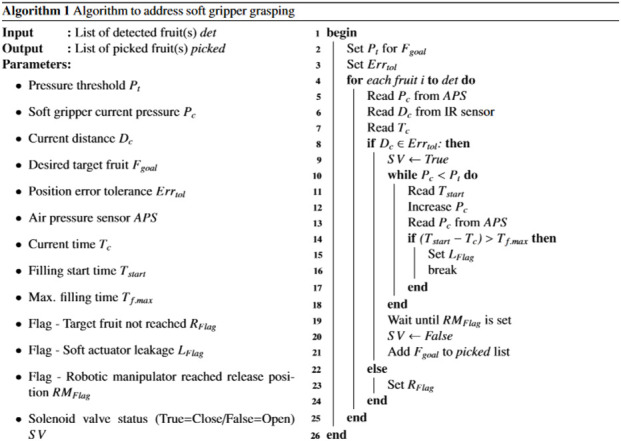

## 4 Characterization and assessment of the soft actuators

**FIGURE 8:**
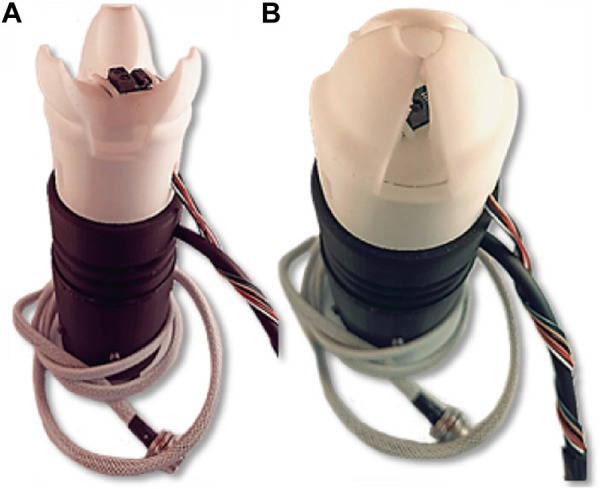
**(A)** Soft Gripper in open position. **(B)** Soft Gripper in closed position.

In order to characterize the soft actuators and ascertain the viability of the proposed approach, a series of experimental tests were conducted. Firstly, various static experiments were carried out to measure the physical characteristics of the actuator. These included the weight of the actuator, the range of fruit diameters that could be manipulated, the maximum opening diameter, and the range of pressure. Figure 8 depicts the gripper in its open and closed positions.

**FIGURE 9:**
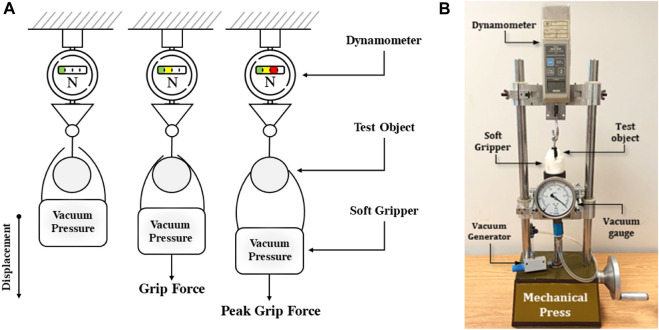
Slip test. **(A)** Experiment schematic view. **(B)** Experiment setup.

Experiments were also conducted to determine the maximum grasping or detachment force of the gripper for a specific geometry at different pressures, which is essential to select the appropriate target fruits for use. This geometry is determined by the target fruit to be manipulated, which in this case was a smooth sphere with a diameter of 20 mm that was printed in PLA using 3D technology. This experiment, commonly known as slip test (Galley et al., 2019) or pull-off force test (Hao et al., 2016), involved setting up a movable element consisting of: (i) the movable base of a mechanical press, (ii) the soft gripper, (iii) fixed elements that included a dynamometer, a pressure measuring device and vacuum generator, and the object to be manipulated. The schematic view and the experiment setup are shown in Figure 9.

The experimental procedure involved varying the position of the soft gripper, which held the object to be manipulated at a predetermined vacuum pressure, while recording the peak force exerted by the object as it slipped off the gripper. This process was repeated while varying the vacuum pressure.

**FIGURE 10:**
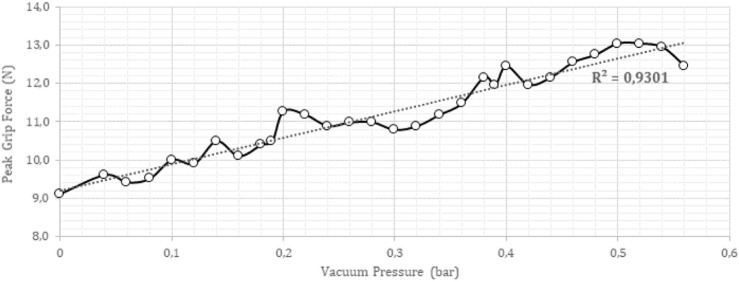
Plot of the slip test for soft gripper characterization showing peak grip force *versus* vacuum pressure.

The results of these experiments are presented in Figure 10, which depicts the relationship between the gripping force and the vacuum pressure exerted by the soft gripper.

As shown in Figure 10, the maximum grasping force of the soft gripper was found to be 13 N at a vacuum pressure of −52 kPa. The gripping force was observed to vary linearly with pressure, with an *R* ^2^ value of 0.93, indicating a strong linear correlation. To ensure the consistency of the results, three soft grippers were tested, and no significant variation was observed among them.

Finally, with all the experiments detailed above, Table 3 summarizes the characteristic values of the designed soft gripper.

**TABLE 3:** Soft gripper characterization.

Soft gripper weight	38.05 g
Weight of the fully assembled gripper	577.55 g
Max. soft actuator diameter (150 kPa)	0.045 m
Min. soft actuator diameter (-52 kPa)	0.007 m
Max. slip force (-52 kPa)	13 N
Operating pressure range	−52–150 kPa
Mean Response Time	≈1 s

**FIGURE 11:**
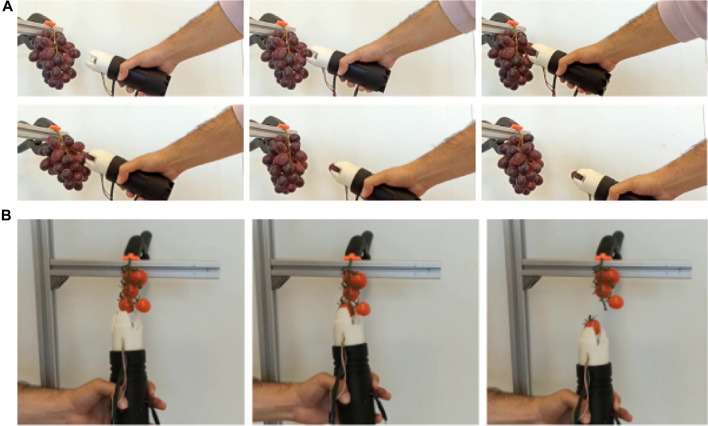
Evaluation of the designed gripper used as a soft tool for picking operations of clustered fruits such as **(A)** grapes and **(B)** cherry tomatoes.

Two other sets of experiments were conducted to evaluate the performance of the soft gripper in picking tasks. In the first set of experiments, the proposed soft gripper is used as a tool manipulated by a human operator. We call this mode of operation soft tool mode. Figure 11 shows several sequences with the proposed gripper in soft tool mode. These experimental tests demonstrate the feasibility of the gripper for pick and place applications, as well as for the harvesting of different fruits in bunches, minimizing the damage caused to the products.

**FIGURE 12:**
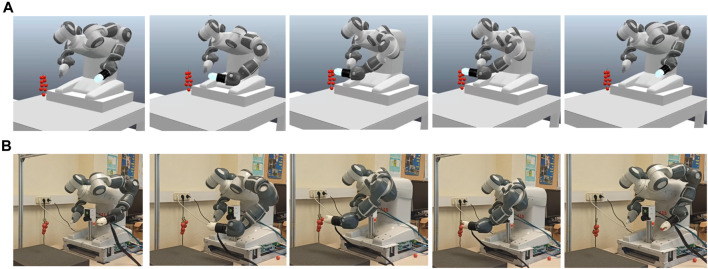
Evaluation of the soft gripper for tomato bunch harvesting operations. **(A)** Simulation of the process in CoppeliaSim. **(B)** Harvest test in laboratory conditions.

In the second set of experiments, an ABB YuMi IRB 14000 dual-arm collaborative robot is used to test the capabilities of the gripper in harvesting tasks (see Figure 12). As the first step of the test, the surface condition of the fruits, which were cherry tomatoes, blueberries, raspberries and grapes, was recorded. The robot and the operation scenario were then simulated in a virtual environment using the CoppeliaSim software. Once the joint positions were obtained in simulation to carry out the movements required for harvesting, the joint coordinates were sent to the robot. As a result of this test, the fruits were successfully harvested without any changes or damage being observed on their surface 1 week after the picking test. It should be noted that although grapes and cherry tomatoes are not typically harvested in this manner, each piece of fruit is picked from a bunch, which represents a challenge in the development of robotic grippers for harvesting (Bachche, 2015). This type of grip is possible thanks to the shape and material used in this soft gripper, which ensures the picking of the selected fruit without damaging the rest of the fruits in the surrounding area.

## 5 Conclusion

The field of soft grippers has seen significant advancements, especially in industrial and medical rehabilitation applications. However, the agricultural sector poses unique challenges, creating opportunities for improvement. This article presents a novel compact hybrid soft gripper design that combines flexible structure with a pneumatic actuation motion suitable as a primary claw or soft tool in robotic manipulators. The proposed design results in a versatile soft gripper, easy to manufacture and assemble, affordable and suitable for unstructured agricultural scenarios, which can harvest small and medium-sized fruits in bunches without damaging the surrounding ones. Moreover, it can also be used in pick and place operations in the food industry.

The design takes advantage of the benefits offered by soft robotics technology. In order to accomplish this objective, the utilization of 3D additive manufacturing has been leveraged, specifically through the use of flexible filaments, with the aim of designing a gripper that integrates two components of soft robotics, flexible structures and pneumatic actuation, in a compact form.

As a future line, there is a need to achieve reliable integration of a variety of sensors into the designed gripper, allowing it to withstand the harsh conditions encountered during the execution of tasks in unstructured environments. Moreover, there is an urgent need to research into the definition of soft gripper joints in ROS to match their actual behavior, in order to facilitate their integration into a next-generation of robot harvesters. The trajectory planning of the ROBOCROP dual-arm robot endowed with the proposed soft gripper for harvesting applications will also be investigated.

## Data Availability

The datasets presented in this study can be found in online repositories. The names of the repository/repositories and accession number(s) can be found in the article/supplementary material.

## Appendix

The 3D printing parameters were optimized using Ultimaker Cura for printing an airtight soft pneumatic bellow gripper. A nozzle temperature of 230°C was used for the bellow actuator with a printing bed temperature of 50°C. The printing speed was set to 20 mm/s for the actuators infill while the outline printing speed was lowered to 10 mm/s, to ensure high-quality exteriors and air tightness. A 20% infill was used for both structures with a perimeter overlap of 30%. Finally, a 0.2 mm layer height with 0.4 mm extrusion width were used for the whole structure.
